# Reading acceleration training changes brain circuitry in children with reading difficulties

**DOI:** 10.1002/brb3.281

**Published:** 2014-09-21

**Authors:** Tzipi Horowitz-Kraus, Jennifer J Vannest, Darren Kadis, Nicole Cicchino, Yingying Y Wang, Scott K Holland

**Affiliations:** Pediatric Neuroimaging Research Consortium, Cincinnati Children's Hospital Medical CenterCincinnati, Ohio

**Keywords:** Children, dyslexia, fluency, imaging, reading

## Abstract

**Introduction:**

Dyslexia is characterized by slow, inaccurate reading. Previous studies have shown that the Reading Acceleration Program (RAP) improves reading speed and accuracy in children and adults with dyslexia and in typical readers across different orthographies. However, the effect of the RAP on the neural circuitry of reading has not been established. In the current study, we examined the effect of the RAP training on regions of interest in the neural circuitry for reading using a lexical decision task during fMRI in children with reading difficulties and typical readers.

**Methods:**

Children (8–12 years old) with reading difficulties and typical readers were studied before and after 4 weeks of training with the RAP in both groups.

**Results:**

In addition to improvements in oral and silent contextual reading speed, training-related gains were associated with increased activation of the left hemisphere in both children with reading difficulties and typical readers. However, only children with reading difficulties showed improvements in reading comprehension, which were associated with significant increases in right frontal lobe activation.

**Conclusions:**

Our results demonstrate differential effects of the RAP on neural circuits supporting reading in both children with reading difficulties and typical readers and suggest that the intervention may stimulate use of typical neural circuits for reading and engage compensatory pathways to support reading in the developing brain of children with reading difficulties.

## Introduction

### Dyslexia: behavioral and neurological characteristics

Developmental reading disability (RD) or dyslexia is characterized by slow, inaccurate reading that cannot be attributed to sensory difficulties, low IQ, or educational deprivation. Reading difficulties typically continue into adulthood despite remedial intervention and repeated exposure to the written language (Fletcher 2009). Individuals with reading disability (RD) experience phonological and orthographic deficits (Share 2004) and exhibit impairments in speed of processing (Breznitz and Misra 2003), rapid automatized naming (Wolf et al. 2000), working memory (Brosnan et al. 2002), and executive function (Helland and Asbjornsen 2000; Brosnan et al. 2002). Recent theories suggest a key role of executive functions in modulating these reading processes for effective and proficient reading ability (Horowitz-Kraus and Breznitz 2008; Horowitz-Kraus 2012; Booth et al. 2013; Kieffer et al. 2014). Several magnetic resonance imaging (MRI) studies confirm that children with RD show atypical structure and function in brain regions related to orthographic and phonological processes, including the left visual cortex, left lateral temporal cortex, and left supramarginal and angular gyri (Breier et al. 2003; McCandliss and Noble 2003; Turkeltaub et al. 2003; van der Mark et al. 2011; Olulade et al. 2012; Yeatman et al. 2013). Despite the altered brain activation in individuals with RD during reading, patterns of functional reorganization have been described previously in this population. It has been suggested that individuals with RD either (1) “compensate” for their reading difficulties by engaging different neural circuits than those of typical readers (TRs) (Simos et al. 2002) or (2) “normalize” by activating similar neural circuits used by TRs (e.g., Rezaie et al. 2011). The purpose of the current study was to determine whether training with an executive-function-based reading program results in a compensation or normalization of the neural circuits related to reading in children with RD, compared to TRs. Understanding the changes following reading acceleration training that encompasses both bottom-up (reading) and top-down (executive functions) elements may pinpoint the crucial neural pathways that we should stimulate in order to achieve the greatest behavioral outcomes (i.e., reading scores) and identify those used by TRs (i.e., normalization) versus those specific to children with RD (i.e., compensation).

### Functional reorganization in children with RD: normalization strategies

The main normalization strategy reported in the literature for individuals with RD and the goal of most interventions for this population, focus on a shift in neural activation from the right brain hemisphere to the left. At baseline, several studies have reported an atypical increase in activation of the right hemisphere during reading, as measured by either functional MRI (fMRI) (Pugh et al. 2000; Shaywitz et al. 2002) or magnetoencephalography recordings (MEG) (Simos et al. 2002). It was suggested that, within the normal course of development, a natural shift of activation occurs from bilateral to the left hemisphere when processing reading materials (Simos et al. 2002; Turkeltaub et al. 2003; Vigneau et al. 2011; Hoeft et al. 2011; Shaul et al. 2012; Dehaene 2013 for review). This shift may be impaired in children with RD and they may overcome this impairment through increased reliance on the right hemisphere when processing written materials (Simos et al. 2002; Hoeft et al. 2011; Dehaene 2013).

Following different reading intervention programs, increased activation in the left hemisphere in children with RD postintervention has been reported. Simos et al. (2002) used a phonologic-based intervention program in children with RD and TRs and detected left-lateralized and more-focused reading-related activation in both groups after intervention (Simos et al. 2002). Krafnick et al. (2011) used a visual-orthographical training in children with RD and TRs and found a bilateral increase in gray-matter volume in regions supporting orthographic processes in children with RD (i.e., the left anterior fusiform gyrus, hippocampus, and precuneus). Such studies provide evidence supporting the idea that effective interventions for RD should be accompanied by some degree of normalization of the brain activity supporting reading.

### Functional reorganization in children with RD: compensation strategies

An alternative, but purportedly effective compensation strategy in children with RD is a reliance on frontal lobe functions encompassing executive functions (Rumsey et al. 1997; Pugh et al. 2000). The authors suggested that at baseline, when children with RD encounter a word that they struggle to decode, the inferior frontal gyrus (related to semantic abilities) and the dorsolateral prefrontal cortex (related to executive functions) are employed as compensatory circuits (see also Heim et al. 2013 for demonstration of this phenomenon in adults). Démonet et al. (2004) also reported a greater activation of frontal regions in children with RD (specifically of the inferior frontal regions) compared to TRs and suggested that this reflects the compensatory pathways of individuals with RD in different types of phonological processing. Heim et al. 2013 suggested that the lower level of activation of the ventral–occipital temporal route for words is shared among individuals with RD. Neuroimaging evidence from these and other studies support the “compensation” hypothesis that effective treatment for dyslexia should stimulate brain activity in new brain regions that are not considered part of the normal reading pathway in TRs.

Given the evidence supporting both compensation and normalization strategies that may be engaged by children with RD, we would anticipate that effective reading interventions have several effects on the brain circuitry for reading. It is possible that children with RD could reinforce compensatory pathways with explicit training on specific neural circuits that are not originally used for reading (compensation). Alternatively, with intensive reading training these children might begin to engage more typical neural circuitry to support improved reading skills (normalization). A third possibility, one that we hypothesize is most likely to occur in the developing brain of a child who is struggling to learn to read, is a mixture of the two strategies in which domain-specific reading intervention in children with RD would increase engagement of typical reading circuits while also enhancing the efficiency of compensatory pathways. In this study, we will test this hypothesis using fMRI, behavioral testing of reading performance, and a computer-based reading intervention program known as the Reading Acceleration Program (or RAP).

### The Reading Acceleration Program

The RAP (Breznitz and Bloch 2010) is a reading fluency program that improves word-decoding accuracy and reading comprehension. This effect was found in both individuals with RD and TRs (Breznitz 1997a,b; Breznitz et al. 2013) and in both young readers (Breznitz 1987, 1992, 1997a,b; Niedo et al. 2013) and adult readers (Breznitz et al. 2013; Breznitz and Leikin 2001; Horowitz-Kraus and Breznitz 2011). Fluent reading depends on accurate, on-time decoding of words (Breznitz 2006) and relies on intact phonology, orthography, and semantics and basic cognitive abilities, such as attention and executive functions (Christopher et al.^2012^). The baseline assumption for the foundation of the RAP is that there is a reciprocal relationship between reading speed, accuracy, and comprehension and as such, a slow pace of reading is an independent causal factor for poor reading. The RAP forces the reader to visually follow the letters (engaging visual attention) as they are erased from the screen (reliance on working memory) at a progressively faster speed (reliance on speed of processing abilities) (Breznitz et al. 2013; Horowitz-Kraus et al. 2013). Monitoring comprehension ensures that the trainees do not only track the letters with their eyes but that they also keep this information in their working memory and process the linguistic information. This procedure forces the reader to circumvent reliance on a slow phonological coding process and therefore to process words in a fast, holistic manner (Breznitz et al. 2013), which “releases” the bottleneck in working memory and enables comprehension (Horowitz-Kraus et al. 2013). In turn, the readers' ability to read words improves as their mental lexicon becomes more stable and their error monitoring improves (Horowitz-Kraus and Breznitz 2013; Horowitz-Kraus et al. 2013, 2014). This was true for both individuals with RD and TRs. The RAP training improves executive functions, such as error monitoring during a lexical decision task (compared to TRs who trained on the RAP) (Horowitz-Kraus et al. 2013) and working memory and attention abilities (as compared to age-matched children with RD who did not train on the RAP) (Niedo et al. 2013), all of which are related to frontal lobe activation. However, since these studies did not employ high spatial resolution imaging tools, the neural circuits involved were not identified and we therefore cannot conclude whether the improved reading and executive functions were related to compensation or normalization pathways.

Based on these studies and building upon our previous findings, in the current study we sought to determine whether training with the RAP results in a compensation or normalization of neural circuits related to reading in English-speaking children with RD and TRs.

In the present study, we sought to test our basic hypothesis that a child who is struggling to learn to read, will use a mixed strategy that will increase engagement of typical reading circuits while also enhancing the efficiency of compensatory pathways. We use functional magnetic resonance imaging (fMRI) to examine remodeling of the neural substrates for reading in children with RD after 4 weeks of training with the RAP. Similar imaging and training is performed in TRs to allow us to demonstrate that the influence of training with the RAP is specific in children with RD. Behavioral reading measures are used to assess the change in reading ability following RAP training in both groups of children. Based on the theory of how the RAP works to improve reading, we can make several predictions about what effect the training will have on reading performance and brain activity patterns in children with RD and TRs. First, we predicted that (1) both children with RD and TRs who train on RAP would show improved reading speed, accuracy, and comprehension as a result of training with the RAP, as demonstrated previously (Breznitz et al. 2013; Horowitz-Kraus et al. 2013; Niedo et al. 2013), but with a greater improvement in children with RD since their starting point in reading proficiency was lower and consistent with previous findings. We postulated that prior to training with the RAP, children with RD would exhibit atypical bilateral activation of regions known to support orthography and phonological processing in TRs. Second, we predicted that (2) following the RAP training, improved reading performance would be supported by increases in left hemisphere activation in these likely reading circuits for both groups (normalization). Due to the executive functions elements implemented in the RAP and the behavioral evidence of improved executive functions following training (Horowitz-Kraus et al. 2013), our third prediction is that (3) increased frontal lobe activation should occur in children with RD (compensation) but not in the TR group. Finally, given that in previous studies the effect of the RAP have shown improvements in contextual reading speed, accuracy, and fluency (Horowitz-Kraus and Breznitz 2013; Horowitz-Kraus et al. 2013), we predicted that the (4) gain in these reading measures, which rely on executive functions and speed of processing, would be significantly correlated mainly with compensation pathways. To test our main hypothesis and these four predictions about the effect of training with the RAP in TRs and children with RD, we conducted a case–control study in children with reading difficulties compared with typical readers during a 4-week intervention trial using RAP and fMRI plus reading testing before and after training. In order to control for the effect of motivation and exposure to the reading tests on reading outcomes, a wait-listed group of children with RD performed the behavioral portion of the study (i.e., reading tasks), but not the fMRI. The effect of the RAP on neural circuits related to reading was examined on a priori regions of interest identified in the literature.

## Materials and Methods

### Participants

Thirty-three children with RD (mean age = 9.9 years, standard deviation [SD] = 1.3 years; 17 females) and 18 TRs (mean age = 9.8 years, SD = 1.7 year; nine females) participated in the current study. Nonverbal IQ scores were determined for all participants using the Test of Nonverbal Intelligence – 3rd edition (TONI-3) (Brown et al. 1997). Nonverbal IQ score was used to ensure that all participants had at least average range IQ and that the two groups were not significantly different for IQ (mean standard score = 103, SD = 7.43). Participants were divided into experimental (*n* = 18 children with RD and 18 TRs) and wait-list groups (*n* = 15 children with RD). Members of the experimental group received the RAP training intervention, whereas the wait-list group has not received the training but had an opportunity to use the RAP upon completion of the study. All participants were native English speakers, right-handed, displayed normal or corrected-to-normal vision in both eyes, and had normal hearing. None had a history of neurological or emotional disorders. Children with RD and TRs were recruited from posted ads and through commercial advertisement. Participants in the TRs group were students of the same chronological age who volunteered for the study and had fluent and accurate reading (according to accepted norms).

Children who were assigned to the wait-list group did not receive the RAP training, but were included in the study to account for possible carryover effects in repeat behavioral assessments as well as the effect of motivation to participate in a research study. Wait-list participants underwent assessment at enrollment and again after 4 weeks (equivalent to the intervention time-frame), but were not scanned. After completion of the second assessment, wait-list participants were invited to participate in the RAP training.

All participants gave their informed written assent and their parents provided informed consents prior to inclusion in the study, and all were compensated for their participation. The Cincinnati Children's Hospital Medical Center (CCHMC) Institutional Review Board approved the experiment. The study was carried out in the imaging center of the CCHMC Pediatric Neuroimaging Research Consortium (PNRC) in Cincinnati, Ohio.

### Reading assessment

Children with RD either received previous diagnoses or parents had reported their children as having reading difficulty (confirmed by the study's reading battery). Reading ability in both children with RD and TRs was evaluated using a battery of normative reading tests in English: (1) untimed single-word reading ability (letter–word subtest from Woodcock-Johnson Tests of Achievement – 3rd edition [WJ-III]; Woodcock and Johnson 1989), (2) untimed pseudoword reading (word-attack subtest from WJ-III), (3) word-reading fluency (Sight Word Efficiency subset to assess word-reading fluency [Test of Word Reading Efficiency – 2nd edition: TOWRE-II]; Torgesen et al. 1999), (4) decoding fluency (Pseudoword Efficiency subset to assess pseudoword decoding fluency from TOWRE-II), (5) reading comprehension to assess understanding of oral reading of connected text (Comprehensive Test of Phonological Processing – 2nd edition [CTOPP-2]; Wagner et al. 1999), and (6) fluency test to assess speed of oral reading of connected text (Gray Oral Reading Test – 4th edition [GORT-IV]; Wiederholt and Bryant 1992). Participants in the RD group had to reach standard scores of −1.5 or below in words reading, decoding, and fluency abilities.

### Attention assessment

Attention was assessed using Conners' Rating Scales – Parent Rating Scales and Self-Report Scales (Conners 1989). These measures were acquired in all participants, and then percentile scores were compared between the groups using independent *t*-tests to verify that the TRs and children with RD in the experimental group were not significantly different for attention ability (self-report *t*_36_ = 1.227, *P *>* *0.05 and parents report *t*_36_ = 0.249, *P *>* *0.05). Both children with RD and TRs in the experimental group underwent baseline behavioral and neuroimaging assessment, 4 weeks of training with the RAP, and follow-up behavioral and neuroimaging assessments.

### Behavioral baseline reading measures

In order to enroll the participants into one of the two reading groups (children with RD vs. TRs), baseline word reading, decoding, and fluency measures (as described in the *Reading assessment* section) were determined (Test 1). The reading measures were used to assess the effects of the RAP (on both children with RD and TRs in the experimental group) as well as silent reading speed and comprehension measures from the RAP evaluation mode. Reading measures were also administered to the wait-list group to eliminate the effects of motivation or exposure to the reading tests on reading scores in children with RD in the experimental group. Each reading assessment lasted approximately 1.5 h.

### Assessment of the reading intervention

#### Behavioral reading assessment

To measure the effects of the RAP on reading ability, we repeated the reading measures described above after 4 weeks of training with the RAP (Test 2).

#### Analysis of behavioral reading measures

To assess the main effects of Group, Test, and the Group × RAP training interaction following the RAP training on different reading levels (silent reading, oral reading, and single word and pseudoword reading), we performed separate repeated measures analyses of variance (RM ANOVAs) on each of the reading measures. Post hoc paired and independent *t*-tests were performed in order to reveal the source of the interaction.

#### Correlation of behavioral reading measures

Since the RAP trains contextual silent reading and the fMRI task employs a single-word recognition paradigm, we correlated the contextual reading scores (oral reading from the RAP and silent reading from the GORT-IV) with word reading from the TOWRE-II battery. A Pearson correlation was performed for the entire sample.

### Neuroimaging assessment

#### Lexical decision task

Both children with RD and TRs in the experimental group (those receiving the RAP training) completed two MRI sessions both before and after the reading training that included alignment and anatomical scans followed by an fMRI paradigm. The effect of the RAP intervention is related to orthographic patterns (i.e., specifically to words; Breznitz 2006; Horowitz-Kraus et al. 2013), a route that can be examined using the lexical decision task (Fiebach et al. 2002). We therefore examined the fMRI results only for words contrasted with pseudowords, focusing on the results of lexical decision-making differences between children with RD and TRs before and after training with the RAP.

Stimuli for the lexical decision task consisted of 12 blocks of text items, either words or pseudowords (modeled after van der Mark et al. 2011), and participants indicated whether the stimuli were real words or not through button-pressing. Word stimuli were 30 high-frequency words (4–6 letters long) matched for imageability and concreteness (adapted from van der Mark et al. 2011). The 30 pseudowords were created by substituting 1 or 2 letters in real words. The stimuli were randomized within each block between the participants and presented horizontally in the center of the screen using DirectRT software (version number 2010.2.103.1115; Empirisoft Corporation, New York, NY). Following the presentation of each word/pseudoword, participants were provided with a response screen containing two faces for either “yes” or “no” responses. The participants were instructed to push the button on the response box using their right hand, corresponding with the “yes” sign, for real words and using their left hand, corresponding with the “no” sign, for pseudoword stimuli. Six blocks of words and six blocks of pseudowords were presented alternately, with five stimuli each (a total of 60 stimuli). Each stimulus was presented for 1600 msec, and after each stimulus, a “yes/no” screen was presented for 1000 msec. The fMRI task lasted 2.6 min (156 sec) for each participant. Practice sessions with 10 stimuli both outside and inside the scanner were performed before the scan session. To avoid priming or anticipation of the stimuli after the first exposure, two different sets of stimuli were used before and after training that were matched for frequency and imageability.

### MRI acquisition

Participants were acclimated and desensitized to condition them for comfort inside the MRI scanner (see Byars et al. 2002 and Vannest et al. in press for details). Head motions were controlled using elastic straps that were attached to either side of the head-coil apparatus used for the scan.

MRI scans were obtained using a 3T Philips Achieva MRI scanner. An MRI-compatible audio/visual system (Avotec, SS3150/SS7100; Avotec, Inc., Buck Hendry Way Stuart, FL) was used for presentation of the stimuli as well as a movie during the preparation (e.g., shimming) and acquisition of the whole-brain anatomical scans. A gradient echo planar sequence was used for T2*-weighted BOLD fMRI scans with the following parameters: TR/TE = 2000/38 msec; BW = 125 kHz; FOV = 25.6 × 25.6 cm; matrix = 64 × 64; slice thickness = 5 mm. Thirty-five acquired slices covered the entire cerebrum. Seventy-eight image volumes were acquired during the fMRI experiment consisting of 30 sec per condition for a total acquisition time of 2 min and 36 sec. A 3D T1-weighted inversion recovery gradient echo anatomical whole-brain scan also was acquired from each participant for anatomical coregistration and for use in spatial normalization of the functional MRI data.

### MRI data analysis

#### Data preprocessing and first level analysis

Using SPM8 (Welcome Department of Cognitive Neurology, London; http://www.fil.ion.ucl.ac.uk/spm/), images were slice-time corrected and realigned. Data were normalized using the 3D anatomical whole-brain scan (7th degree-spline interpolation) to match the Montreal Neurological Institute (MNI) standard template, resampled (3 mm^3^ voxels), and smoothed with 8-mm full width at half maximum (FWHM). A general linear model approach was used to identify voxels activated by the task for each participant. The second level analysis was based on the individual contrast images (words > pseudowords).

We tested our hypotheses regarding normalization versus compensation neural strategies by focusing on a set of a priori selected regions of interest (ROIs) that were previously reported to show activation during word reading. Ten (10) of these regions were derived from meta-analyses of reading (Bolger et al. 2005; Horowitz-Kraus and Breznitz 2013; Houde et al. 2010; Koyama et al. 2011; Richlan et al. 2009). We also include an ROI in the anterior cingulate cortex (ACC) (Horowitz-Kraus and Breznitz 2013) because of its role in executive function. The 11 ROIs included regions related to the orthographic/visual stream: (1) inferior occipital gyrus [IOG (BA)18], (2) posterior fusiform gyrus (FFG, BA 37); phonological processing, (3) posterior superior temporal gyrus (STG, BA 41), (4) temporoparietal junction (TPJ, BA 22), (5) inferior parietal lobule (IPL, BA 40), (6) intraparietal sulcus (IPS, BA 7), (7) dorsal precentral gyrus (PCG, BA 4); semantic processing, (8) inferior frontal gyrus pars opercularis (IFGop, BA 44), (9) inferior frontal gyrus pars triangularis (IFGtr, BA 46); and executive control, (10) middle frontal gyrus (MFG, BA 9), (11) ACC, BA 32. All regions were inspected bilaterally. Each ROI was a spherical seed (6 mm radius in 2 mm standard space) (Koyama et al. 2011) centered on the MNI coordinates (adapted from Koyama et al. 2013; Koch et al. 2011). The ROIs and MNI coordinates are listed in Table 1 and referred to in subsequent analysis sections and the Results. Since the current study focused on these specific ROIs, all imaging data were extracted from these a priori selected ROIs. Our analysis approach is divided into purely imaging analysis and imaging data correlated with behavioral data, as an attempt to explore the interactions between gain in behavioral reading measures and change in activation in the selected ROIs.

**Table 1:** Regions of interest (ROIs) and Montreal Neurological Institute (MNI) brain coordinates.

Related cognitive ability	Region of interest		BA	*X*'	*Y*'	*Z*'
Orthographical processing	IOG—inferior occipital gyrus	Left	18	−25	−87	−10
		Right		25	−87	−10
	FFG—fusiform gyrus (posterior)	Left	37	−48	−57	−20
		Right		48	−57	−20
Phonological processing	STG—superior temporal gyrus	Left	41	−53	−31	9
		Right		53	−31	9
	TPJ—tempo-parietal junction	Left	22	−59	−45	15
		Right		59	−45	15
	IPL—inferior parietal lobule	Left	40	−40	−48	42
		Right		40	−48	42
	IPS—inferior parietal sulcus	Left	7	−30	−58	48
		Right		30	−58	48
Motor function	PCG—precentral gyrus (dorsal)^1^	Left	4	−48	−12	45
		Right		48	−12	45
Semantic processing	IFGop—inferior frontal gyrus (opercularis)^2^	Left	44	−51	10	10
		Right		51	10	10
	IFGtr—inferior frontal gyrus (triangularis)^2^	Left	46	−48	32	6
		Right		48	32	6
Executive functions	ACC—anterior cingulate cortex	Left	32	−8	39	9
		Right		8	39	9
	MFG—middle frontal gyrus	Left	9	−44	10	30
		Right		44	10	30

BA, Broca's area.

1 PCG is also part of phonological processing (Houde et al. 2010).

2 IFGtr and IFGop are also part of executive functions (see also Horowitz-Kraus et al. 2014).

### Second level analysis (MRI group composites before and after training with the RAP)

For each ROI, group-level analyses were carried out using a random effects ordinary least-squares model. Small-volume correction (i.e., voxel-wise analysis) for multiple comparisons was performed using Gaussian Random Field Theory (min Z > 3; cluster significance: *P *<* *0.05, FWE corrected). To test the effect of training in the two reading groups, we performed a two-way ANOVA, treating Group as a two-level factor (children with RD and TRs) as well as Training with the RAP (Test 1 = baseline, Test 2 = 4-week follow-up). The ANOVA and pairwise comparisons were performed on the beta values of the main effect variable (beta value) for the contras of words > pseudowords in each region, with a small-volume correction for multiple comparisons. This approach takes into account all of the voxels in the ROI and the small-volume correction is done across those.

### Interrogation of pairwise group differences

Subsequently, regions exhibiting significant main effects of the Group or Training with the RAP (i.e., the effect of “Test”) or that showed a significant Group × Training with the RAP interaction, were interrogated by post hoc analysis using independent *t*-tests to determine significant pairwise differences between groups and paired *t*-test between Tests 1 and 2. Bonferroni correction was used at the ROI level to control for multiple comparisons.

### Correlations of gain in reading scores and change on ROI activation

In the same set of ROIs, we used multiple regressions to explore relationships between the activation in each region after training with the RAP and the magnitude of gains in reading performance (i.e., the difference between the scores in Tests 1 and 2). Since the RAP improves reading speed, accuracy, and fluency (Horowitz-Kraus and Breznitz 2013; Horowitz-Kraus et al. 2013), we examined this relationship separately for each of the three following scores: (1) contextual reading rate (GORT-IV), (2) contextual reading accuracy (GORT-IV), and (3) words/pseudowords reading fluency (TOWRE-II) that was measured by the gain in efficiency score for these two measures.

### Reading acceleration program

#### Stimuli

The RAP bank of 1500 sentences is composed of moderate-to-high frequency of words in the English language (http://www.wordfrequency.info/). Each stimulus is a sentence with a multiple-choice question followed by four possible answers. Each sentence length is 9–12 words, comprised 45–70 letters, letter width of 5 mm, extending over 1 to 2 lines and with 18 mm between lines. Each sentence is presented once during the entire training intervention. The RAP sentences have been tested and verified for their level of difficulty in previous studies (Breznitz 2006; Breznitz et al. 2013; Horowitz-Kraus et al. 2013).

#### Training procedure

Reading training was administered via the internet using a computer in the participant's home. The participants' compliance was monitored by a remote access to the training records, with verification of the record of five training sessions per week. Only datasets of participants who completed at least 18 total training sessions were included in the study. The participants were trained for 4 weeks, five times each week at 15–20 min per session for a total of 20 sessions, and reading a different set of 50 randomly presented sentences in each session. The initial and the final reading pace and comprehension were measured by the evaluation mode of the RAP, which measures these variables in a self-paced reading condition (Breznitz et al. 2013).

The duration of a sentence display on the screen was calculated individually for each participant based on the evaluation mode and was controlled by text erasure, starting from the beginning of the sentence and advancing at a given per-character rate. All participants were presented with the same sets of sentences, in the same order. They were instructed to read the sentence silently and while doing so, the sentence disappeared from the computer screen and a multiple-choice comprehension question appeared and remained on the screen until the participant responded. They were instructed to choose the correct answer by pushing the corresponding number on the numeric keypad of the computer. The disappearance of the question from the computer screen prompted appearance of the next sentence.

#### Presentation rate and evaluation mode

The initial text erasure rate was determined specifically for each participant, based on a pretest evaluation mode administered prior to training. The evaluation mode consists of 12 sentences and 12 multiple choice questions (Breznitz and Leikin 2000). The sentences in the evaluation mode remained on the screen until the participants finished reading them. The participants were instructed to read the sentences silently and to push the space button on the keyboard when finished reading, which prompted a comprehension question. The mean reading rate (msec per letter) for the sentence correctly answered determined the initial presentation rate of the RAP for that participant.

#### Accelerated training condition

The initial reading rate in the RAP training mode is determined based on the reading rate calculated in the evaluation mode (based on the reading rate of 12 sentences). In the first training session, 50 sentences were presented consecutively on the screen. The letters in each sentence disappeared one after the other, according to the mean reading time (msec per letter) recorded on the pretest. Following the disappearance of the sentence from the computer screen, participants were instructed to answer a comprehension question. The per-letter “presentation rate” decreased from one sentence to the next in by 2% for each subsequent sentence (Breznitz 1997a,b) and the “disappearance rate” increased only when the participants' answers to the probe questions were correct on 10 consecutive sentences. In other words, the computer pacing is modified periodically based on participant performance with the goal of increasing the pace over what would be chosen by the participant.

## Results

### Baseline reading measures

Results of *t-*tests between participants in the RDs and TRs groups, and between the wait-list and experimental (trained) RDs groups at baseline (Test 1) revealed no significant differences in IQ or attention measures (see Table 2 for comparison of children with RD in the experimental and wait-list groups and Table 3 for comparison between children in the RD and TRs in the experimental group). Also, no differences in reading ability were found between children with RD in the experimental and children with RD in the wait-list groups (Table 2, Test 1 results). However, children with RD read significantly slower and less accurately than TRs (Table 3, Test 1 results).

**Table 2:** Reading measures in children with RD who either received the Reading Acceleration Program intervention or were enrolled to the wait-list group, both before (Test 1) and after (Test 2) training with the RAP.

	Test 1		Test 2		*t*-test	Contrasts
Experimental (A)	Wait-list (B)	Experimental (C)	Wait-list (D)			
Age (years)	9.9 (1.2)	9.9 (1.7)	–	–	ns	–
IQ (TONI-3, in percentile)	100 (8.9)	102.2 (10.3)	–	–	ns	–
Word reading fluency (TOWRE-II, in percentile)	13.26 (15.88)	12.2 (10.8)	27.69 (17.09)	11.33 (10.1)	−3.384**	C > A
					2.046*	C > D
Pseudoword reading fluency (TOWRE-II, in percentile)	15.41 (12)	10 (5.4)	25.07 (17.52)	9.2 (6.9)	−2.74*	C > A
					2.041*	C > D
Contextual oral reading rate (GORT-IV, in percentile)	10.5 (8.17)	14.29 (10.45)	24.83 (12.73)	14.21 (12.04)	−6.442***	C > A
					1.816*	C > D
Contextual oral reading accuracy (GORT-IV, in percentiles)	15.15 (12.11)	16.47 (10.6)	24.5 (7.46)	16.27 (13.34)	−4.3***	C > A
Oral reading comprehension (GORT-IV, in percentile)	21.95 (7.42)	26.36 (19.85)	36.53 (12.09)	33.57 (21.89)	−3.596**	C > A
Phonological awareness (CTOOP, “Elision” subtest, in percentile)	22.75 (23.93)	23.6 (21.88)	31.9 (28.8)	25.13 (26.05)	−2.082*	C > A

IQ, TONI-3, Test of Nonverbal Intelligence – 3rd edition; TOWRE-II, Test of Word Reading Efficiency – 2nd edition; GORT-IV, Gray Oral Reading Test – 4th edition; CTOOP-2, Comprehensive Test of Phonological Processing – 2nd edition.

Mean (standard deviation) for individuals with RD in the experimental group (received the Reading Acceleration Program intervention) versus those in the wait-list group on reading measures. The *t*-test column represents the data from the paired and independent *t*-test analyses

* *P *<* *0.05;

** *P *<* *0.01;

*** *P *<* *.001.

Ns = no significant differences between the conditions. The contrasts column represents the relationship between the measures in the paired *t*-test (A vs. C and B vs. D) and independent *t*-test analyses (A vs. B and C vs. D).

**Table 3:** Reading measures for both children with RD and TRs who received the Reading Acceleration Program intervention, both before (Test 1) and after (Test 2) the training.

	Test 1		Test 2		*t*-test	Contrasts
Children with RD (A)	TRs (B)	Children with RD (C)	TRs (D)			
Age (years)	9.9 (1.2)	9.8 (1.68)	–	–	ns	–
IQ (TONI-3, in, percentile)	100 (8.9)	104.58 (6.82)	–	–	ns	–
Word reading fluency (TOWRE-II, in percentile)	13.26 (15.88)	56.37 (22.74)	27.69 (17.09)	75.74 (16.43)	−3.384**	C > A
					−6.772***	B > A
					−5.327***	D > B
Pseudoword reading fluency (TOWRE-II, in percentile)	15.41 (12)	59 (20.4)	25.07 (17.52)	74.32 (19.58)	−2.74*	C > A
					−8.011***	B > A
					−4.669***	D > B
Contextual silent reading rate (RAP, in msec/letter)	166.3 (60.31)	102.84 (38.59)	125.91 (44.10)	72.81 (19.39)	2.321*	A > C
					4.32***	B > D
					3.926***	A > B
					4.821***	C > D
Contextual silent reading comprehension (RAP, in msec/letter)	64 (6.97)	96.15 (5.28)	88.37 (7.2)	95.67 (6.26)	−10.458***	C > A
					−16.16***	A > B
					−3.366*	C > D
Contextual oral reading rate (GORT-IV, in percentile)	10.5 (8.17)	56.26 (20.19)	24.83 (12.73)	67.21 (15.3)	−6.442***	C > A
					−9.18***	B > A
					−4.319***	D > B
Contextual oral reading accuracy (GORT-IV, in percentile)	15.15 (12.11)	62.89 (23.26)	24.5 (7.46)	70.21 (22.14)	−4.3***	C > A
					−7.978***	B > A
Oral reading comprehension (GORT-IV, in percentile)	21.95 (7.42)	70.47 (16.5)	36.53 (12.09)	76.05 (16.92)	−3.596**	C > A
					−9.529***	B > A
Phonological awareness (CTOOP, “Elision” subtest, in percentile)	22.75 (23.93)	70.63 (16.03)	31.9 (28.8)	72.47 (20.72)	−2.082*	C > A

IQ, TONI-3, Test of Nonverbal Intelligence – 3rd edition; TOWRE-II; Test of Word Reading Efficiency – 2nd edition; RAP, Reading Acceleration Program; GORT-IV, Gray Oral Reading Test – 4th edition; CTOPP-2, Comprehensive Test of Phonological Processing – 2nd edition.

Mean (standard deviation) of reading measures for children with RD versus TRs receiving the reading intervention. The *t*-test column represents the data for the paired and independent *t*-test analyses

* *P *<* *0.05,

** *P *<* *0.01,

*** *P *<* *0.001.

ns = no significant differences between the conditions. The contrasts column represents the relationship between the measures in the paired *t*-test (A vs. C and B vs. D) and independent *t*-test analyses (A vs. B and C vs. D).

### Effect of the RAP on behavioral and neuroimaging measures

The effect of training with the RAP on reading ability was measured using several 2 × 2 (Group × Training with the RAP) RM-ANOVAs:

1. Effect of the RAP on silent reading speed and comprehension (data derived from the evaluation mode of the RAP).

*Speed:* Main effects of Training with the RAP (*F*_1,37_ = 13.482, *P *<* *0.01, *η*^2^ = 0.267) and Group (*F*_1,37_ = 34.226, *P *<* *0.001, *η*^2^ = 0.481) indicating faster reading speed after training with the RAP and a generally slower reading pace in children with RD compared to TRs.

*Comprehension:* Main effects of the Training with the RAP (*F*_1,37_ = 69.133, *P *<* *0.001, *η*^2^ = 0.651) and Group (*F*_1,37_ = 171.878, *P *<* *0.001, *η*^2^ = 0.669) indicating greater comprehension after training with the RAP and lower reading comprehension scores in children with RD compared to TRs. The significant Group × Training with the RAP interaction (*F*_1,37_ = 74.81, *P *<* *0.001, *η*^2^ = 0.828) indicates a greater change following training in comprehension scores in children with RD as compared to the TRs (Table 3). There were no significant differences between Test 1 and Test 2 measures in the children with RD in the wait-list group (control group; children with RD who did not train with the RAP) by *t*-test analysis.

2. Effect of the RAP training on oral contextual reading speed and comprehension (data derived from the GORT-IV).

*Speed:* Main effects of Training with the RAP (*F*_1,37_ = 56.038, *P *<* *0.001, *η*^2^ = 0.602) and Group (*F*_1,37_ = 34.226, *P *<* *0.001, *η*^2^ = 0.481) indicating faster reading speed after the RAP training and a generally slower reading pace in children with RD compared to TRs.

*Comprehension:* Main effects of Training with the RAP (*F*_1,37_ = 13.392, *P *<* *0.001, *η*^2^ = 0.226) and Group (*F*_1,37_ = 118.2, *P *<* *0.001, *η*^2^ = 0.762) indicating greater comprehension after training with the RAP in both groups and generally lower comprehension in children with RD (see Table 3 for results).

3. Effect of the RAP training on word and pseudoword reading (data derived from the TOWRE-II).

*Word reading:* Main effects of Training with the RAP (*F*_1,36_ = 25.333, *P *<* *0.001, *η*^2^ =  0.413) and Group (*F*_1,36_ = 78.829, *P *<* *0.001, *η*^2^ = 0.686) indicating greater reading scores after training with the RAP and lower word-reading scores in children with RD compared to TRs.

*Pseudoword reading:* Main effects of Training with the RAP (*F*_1,36_ = 16.092, *P *<* *0.001, *η*^2^ = 0.309) and Group (*F*_1,36_ = 86.803, *P *<* *0.001, *η*^2^ = 0.762) indicating greater pseudoword reading scores after training with the RAP in both groups and generally lower scores in children with RD compared to TRs (Table 3). No significant effects were found in the wait-list of children with RD (i.e., control group, those who did not receive the RAP training). Since this group did not receive imaging, they were not examined further in the ROI-based image analysis.

#### Correlation of behavioral reading measures

Since the RAP trains contextual silent reading and the fMRI task employs a single-word recognition paradigm, we correlated the contextual reading scores (oral reading from the RAP and silent reading from the GORT-IV) with the scores of the word reading task from TOWRE-II. A Pearson correlation was performed for the entire sample. The analysis revealed that word-reading scores were positively correlated with contextual reading rate (GORT-IV) (*r* = 0.807, *P *<* *0.001), accuracy (*r* = 0.755, *P *<* *0.001), and comprehension (r = 0.758, *P *<* *0.001). Our results suggest that greater word-reading ability is associated with reading comprehension and more accurate, faster contextual oral reading. This is consistent with previous results highlighting the correlation of these skills in children (Berninger et al. 2006).

### MRI data analysis

#### MRI group composites before and after intervention

**Figure 1:**
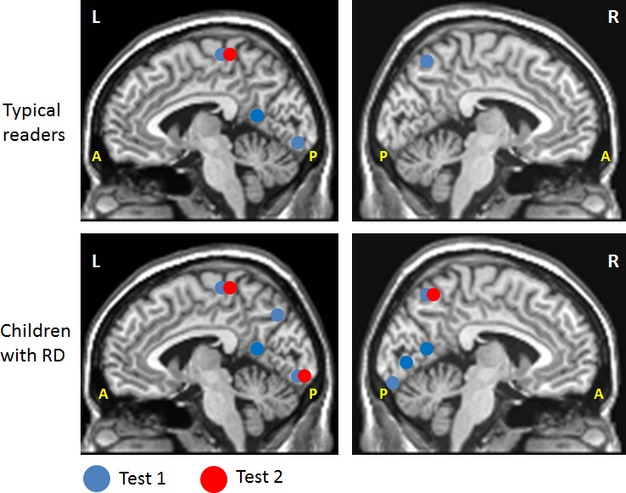
Independent *t*-test analyses for children with RD and TRs before and after training with the RAP (contrast: words > pseudowords). Upper part: Composite maps for TRs before (blue) and after (red) intervention. Lower part: Composite maps for children with RD before (blue) and after (red) intervention. Note: The figures are in neurological orientation (L = left, R = right, A = anterior, P = posterior). Data are significant at *P *<* *0.05, corrected.

To examine the differences in BOLD-signal in the selected ROIs between the children with RD and TRs before and after intervention, independent *t*-test analyses (words > pseudowords) for children with RD and TRs for Tests 1 and 2 were performed. Results demonstrate that before intervention (Test 1, blue dots) the left PCG, STG, IOG, and right IPL were activated in TRs, and after intervention only the left PCG was significantly activated (Test 2- red dots) (Fig. 3, upper part). Children with RD showed activation in the left STG, IOG, IPL, PCG, and right IOG, STG, FFG before intervention (Test 1 – blue dots) and in the left PCG, IOG and right IPL after intervention (Test 2- red dots) (Fig. 1, lower part).

#### Group, training with RAP and interaction effects

**Figure 2:**
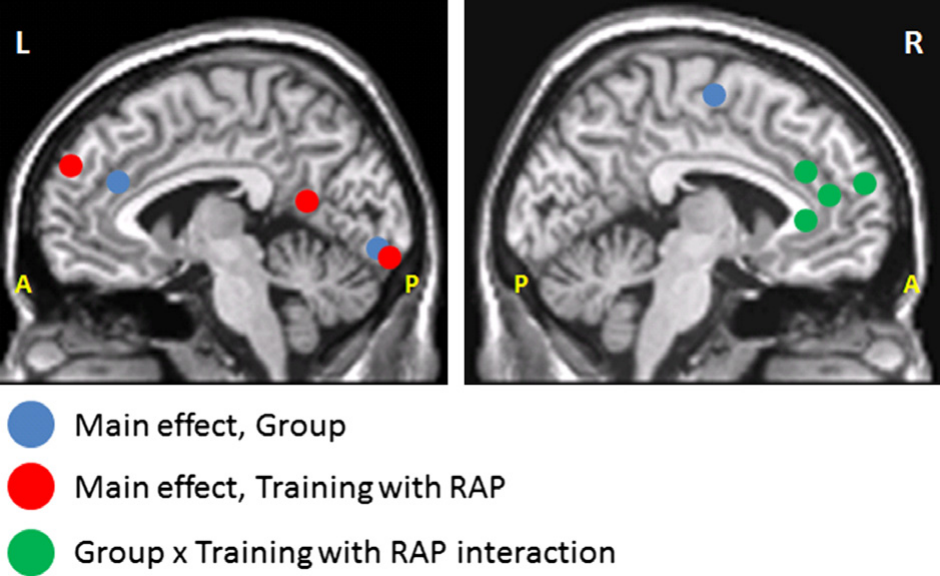
Significant regions of interest for the repeated measures ANOVA (contrast: words > pseudowords). Significant regions of interest (ROIs) for Group × Training with the RAP repeated measures ANOVA. Main effects of training with the RAP (red), Group (blue), and Group × Training with the RAP interaction (green) are marked. Note: The figure is in neurological orientation (L = left, R = right, A = anterior, P = posterior).

A two-way ANOVA, with Group (two levels: Children with RD and TRs) and Training with RAP (two levels: Test 1 and Test 2) for each chosen ROI was performed using small-volume correction (Fig.2). Specifically, we observed a significant main effect of Group (*F*_1,68_ = 8.418, *P *<* *0.05; FWE corrected) in the left and right inferior occipital gyrus (lIOG and rIOG, BA 18), left inferior frontal gyrus triangularis (IFGtr, BA 46), and right precentral gyrus (rPCG, BA 4). A significant main effect of Training with the RAP (*F*_1,68_ = 7.09, *P *<* *0.05; FWE corrected) was found in the left occipital gyrus (lOG, BA 18), left superior temporal gyrus (lSTG, BA 41), and left medial frontal gyrus (lMFG, BA 9). A significant Group × Training with the RAP interaction (*F*_1,68_ = 8.418, *P *<* *0.05; FWE corrected) also was found in the right anterior cingulate cortex (rACC, BA 32), right medial frontal gyrus (rMFG, BA 9), right inferior frontal gyrus opercularis (rIFGop BA 44), and right inferior frontal gyrus triangularis (rIFGtr, BA 46). No activation was found for pseudowords > words contrast.

#### Interrogation of pairwise group differences

Post hoc analysis (i.e., pairwise comparisons via independent and paired *t*-tests) of regions exhibiting a main effect of Group, Training with the RAP, and Group × Training with the RAP interaction revealed that the main effect of Group resulted from the greater activation in children with RD in the lIOG and rIOG (BA 18), rPCG (BA 4), and lIFGtr (BA 46). The main effect of Training with the RAP was attributed to greater activation of the lIOG (BA 18) and the lSTG (BA 41) after intervention (Test 2) and the lMFG (BA 9) before intervention (Test 1). The interaction resulted from a greater activation in the rACC (BA 32) in children with RD in Test 2 versus Test 1, greater activation in the rIFGop (BA 44) and IFGtr (BA 46) in children with RD than TRs in Test 2, and greater activation in the rMFG (BA 9) in children with RD than TRs in Test 1.

#### Correlations of gain in reading scores and activation within the ROIs

**Figure 3:**
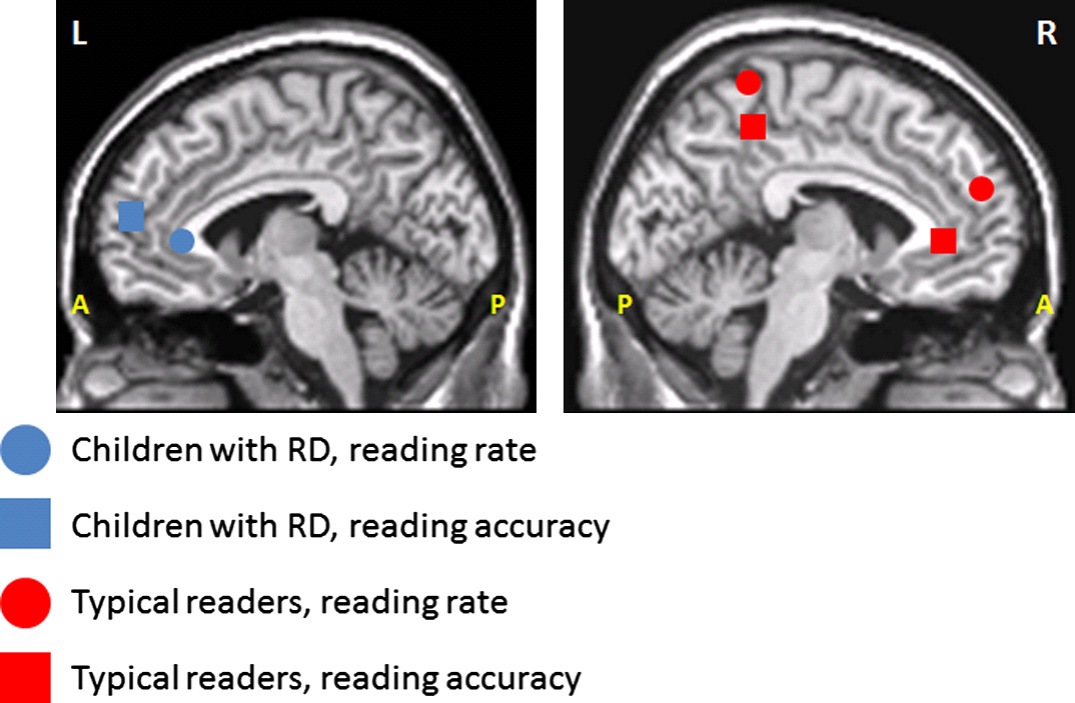
Regression of regions of interest with contextual oral reading speed and accuracy (from GORT-IV) (contrast: words > pseudowords). Positive correlation between activation in regions of interest (ROIs) and the gain in contextual reading speed and accuracy (in percentile) after training with the RAP. Significant correlation between activation in ROIs during reading after intervention (Test 2) and gain in contextual reading speed (in circles) and accuracy (in squares) for children with RD (blue) and TRs (red). Note: The figure is in neurological orientation (L = left, R = right, A = anterior, P = posterior).

**Figure 4:**
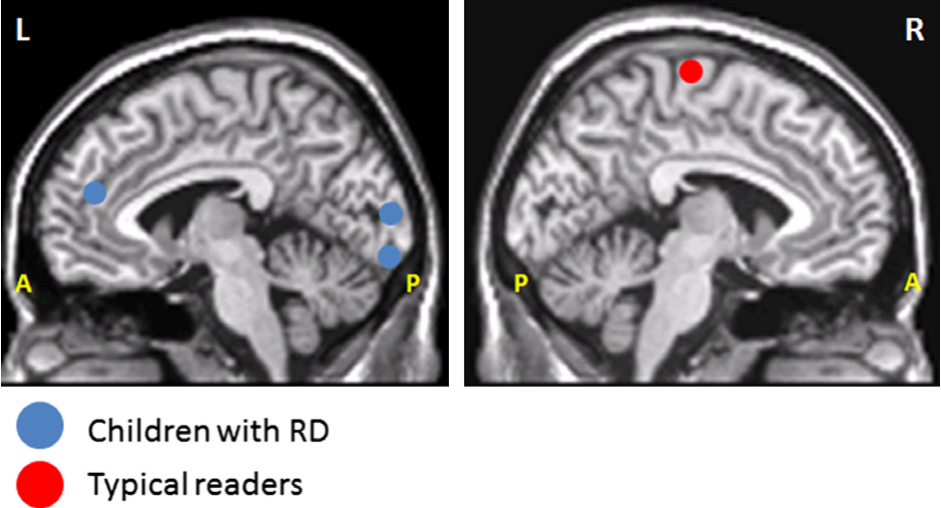
Positive correlation between activation in regions of interest and the gain in efficiency scores from word/pseudoword reading (TOWRE-II, in percentile) after training with the RAP. Significant correlation between activation in regions of interest (ROIs) during reading after training with the RAP (Test 2) and the gain in word/pseudword reading (TOWRE-II). Significant ROI for in children with RD (blue) and TRs (red). Note: The figure is in neurological orientation (L = left, R = right, A = anterior, P = posterior).

In this analysis, we correlated the beta values (linear regression coefficient of the main effect for contrast between conditions) across all voxels in each ROI from Test 2 with the gain in reading scores (the difference between Test 1 and Test 2) for contextual reading rate, contextual reading accuracy (GORT-IV), and word/pseudowords fluent reading (TOWRE-II). After small-volume correction, the following significant correlations were found (Figs3 and 4):

*Children with RD*: Children with RD showed significant positive correlations between the gain in scores for contextual reading rate (GORT-IV) and the activation of the lACC (BA 32), and between the scores for the gain in contextual reading accuracy (GORT-IV) and the activation of the lMFG (BA 9). Significant positive correlations also were found between word/pseudoword efficiency reading scores and the activation of the lMFG (BA 9), lFFG (BA 37), and lIOG (BA 18) (*P *<* *0.05; FWE corrected).

*Typical readers*: TRs showed significant positive correlations between gain in scores for contextual reading rate (GORT-IV) and the activation of the rMFG (BA 9) and rIPL (BA 40), and between the scores for the gain in contextual reading accuracy (GORT-IV) and the activation of the rIFGtr (BA 46) and rIPL (BA 40). Significant positive correlations also were found between word/pseudoword efficiency reading scores and the activation of the rIPL (BA 40) (*P *<* *0.05; FWE corrected).

No significant correlations between the levels of activation in the ROIs in Test 2 and rate/comprehension measures from the RAP were found.

## Discussion

The current study aimed to discover whether children with RD in the experimental group showed reorganization in neural pathways related to reading making them similar to TRs (i.e., “normalization”) or alternative neural patterns that differed from TRs (i.e., “compensation”). Another aim of our study was to explore the effect of the RAP training on neural circuits underlying reading in TRs.

Our results indicate that both children with RD and TRs exhibited improvements in oral and silent reading following 4 weeks of training with the RAP, which is consistent with our hypothesis. We found that children with RD demonstrated faster oral and silent reading, greater comprehension scores as well as more accurate reading following intervention. These findings were not observed in our wait-list group of children with RD, allowing us to infer that the effect was due to the RAP training rather than a placebo effect associated with enrolling in a research protocol. These results confirm previous findings of the positive effect of the RAP in children with RD (Horowitz-Kraus and Breznitz 2013; Breznitz et al. 2013; Horowitz-Kraus et al. 2013; Niedo et al. 2013). We also found increased brain activation in key areas representing both normalization and compensatory brain reorganization to support these improvements in reading skills. Increased brain activation occurred while reading words versus pseudowords in predefined ROIs in the left occipito-temporal and frontal lobes, which supports our hypotheses.

### “Normalized” neural circuits in children with RD following the RAP intervention

We demonstrated that at baseline, children with RD showed greater bilateral occipital (BA 18) and frontal (BA 4, BA 46) activation than TRs. The absence of left hemisphere specialization in reading and particularly the lack of focal activation in the occipital regions may represent the neural correlates of dyslexia (Shaywitz et al. 2002).

More focal activation of left reading regions was found in both groups following training with the RAP, thus supporting the “normalization” theory that proposes this same pattern. More specifically, children with RD showed bilateral activation before training with the RAP (right IOG, STG, FFG, and left STG, IOG, IPL, PCG), which shifted primarily to the left after the training (left PCG, IOG, and right IPL). TRs also demonstrated a greater activation in the left PCG after training. The activation of the regions related to orthographical processing (occipito-temporal) were previously found to be more active during exposure to words (compared to false fonts) in both children and adults (Olulade et al. 2012), while this activation is disrupted in dyslexia (van der Mark et al. 2011). Our findings of greater activation in this region of the left hemisphere after training in children with RD, together with reading improvement, validates studies supporting the resulting “normalization” pathways in children with RD following reading intervention. This reading improvement also might be achieved in combination with the activation of the lPCG following training. The PCG plays an important role in articulation and phonologic retrieval (Turkeltaub et al. 2003; Mechelli et al. 2005; Carreiras et al. 2007; Houde et al. 2010). Therefore, increased activation of this region can support improvement not only in word recognition skills, but also in phonological and articulation skills. This is consistent with our findings demonstrating a relationship between the gain in oral reading scores and MRI data after intervention.

Correlation analysis results also demonstrated greater left lateralization in children with RD following intervention. Specifically, positive correlations were found for the following comparisons: gain in word/pseudoword reading scores and the activation of the left MFG, FFG and IOG; contextual reading rate gains and the lACC; and reading accuracy and the lMFG. TRs showed positive correlation between word/pseudoword reading improvement and the activation of the rIPL and between contextual rate and accuracy with greater activation of the right MFG and IPL and the right IFGtr and IPL, respectively. These seemingly incongruous results in the right hemisphere lead us to further consider the specific effect of the acceleration manipulation on the activation in the right hemisphere.

In the current study, despite a general trend of increased activation in the left hemisphere following training (i.e., “normalization”), children with RD still showed activation in the right hemisphere during reading, especially in the rIPL. Surprisingly, TRs also showed positive association between the activation of the rIPL and greater reading gain after training with the RAP. The rIPL is primarily involved in phonological processes (Meschyan and Hernandez 2006), but also contributes to verbal working memory (LaBar et al. 1999). We therefore postulate that while the rIPL may be active in children with RD due to greater phonological abilities, TRs may activate these regions as a result of improved working memory following training with the RAP, an intervention focused on working memory and speed of processing (Breznitz and Share 1992). Increased working memory and associated brain activation in TRs following training with the RAP may explain the improved contextual reading scores of TRs, as these working memory increases may support better contextual comprehension (see Rimrodt et al. 2008). Further research specifically examining these abilities after administration of the RAP should be conducted to verify this point.

Another possibility is that the remaining activation of the right hemisphere following the RAP in children with RD and the “newly”-formed right-lateralized activation in TRs may be specific to the acceleration manipulation. Involvement of the right hemisphere in accelerated reading was reported by Benjamin and Gaab (2012). This study found greater activation in the right lingual/fusiform and the inferior frontal lobe, during fluent sentence reading than letter reading. As was suggested by Benjamin, these regions respond differently as the ability to read fluently is manipulated, which may explain the increased activation of the right anterior/frontal regions in children with RD following the acceleration manipulation.

### “Compensatory” neural pathways in children with RD following training with the RAP

The RAP resulted in activation of “compensatory” regions as well, with greater frontal activation (BA 4) following training observed in both children with RD and TRs. This activation might be related to the postulated effect of the RAP on working-memory abilities (Breznitz and Share 1992; Horowitz-Kraus and Breznitz 2011, 2013; Horowitz-Kraus et al. 2013). This “compensatory” mechanism suggests that greater reading speed leads to improved decoding and comprehension (Breznitz and Leikin 2000), and acceleration is thought to cause graphemic information to be processed differently than at a slower reading speed (Karni et al. 2005), perhaps resulting in more efficient access to the mental lexicon and more efficient automatic word recognition (Breznitz et al. 2013). This supposition is supported by the greater activation of BAs 40 and 18 in children with RD, which demonstrated increased activation in our study. We also found greater activation in bilateral IFG following training with the RAP in children with RD, which corresponds with improved reading skills as reported in Table 2. The greater activation of frontal regions following acceleration manipulation also has been reported in adult TRs (Benjamin and Gaab 2012). As mentioned, the role of the frontal lobe as a possible compensatory pathway in children with RD by means of semantic retrieval or reliance on executive functions was previously suggested (Rumsey et al. 1997; Pugh et al. 2000). This is in agreement with theories that describe the role of the right frontal lobe in attention recruitment and working-memory engagement (see Vigneau et al. 2011 for a meta-analysis) and may explain the improvement in reading following attention-training video games in individuals with RD (Bavelier et al. 2013). Moreover, a recent study showed a positive association between the greater diffusivity values in the frontal portion of the right arcuate fasciculus, a white-matter tract related to reading (Yeatman et al. 2013), and reading comprehension scores (Horowitz-Kraus et al. 2014). It may be that children with RD in the current study compensated for their reading difficulty by relying on brain structures in the right frontal lobe that support sematic knowledge and comprehension. Future diffusion tensor imaging (DTI) studies that examine the change in diffusivity in the white-matter tracks before and after training with the RAP should verify this point.

Another frontal region related to executive processes, mainly attention and error monitoring, is the ACC. The right ACC (BA 32) activation after training with the RAP in children with RD may be due to greater error monitoring (Horowitz-Kraus and Breznitz 2008; Horowitz-Kraus and Breznitz 2011), which was previously found to be positively correlated with reading level in children with RD (Horowitz-Kraus and Breznitz 2011; Horowitz-Kraus and Breznitz 2013). Since the RAP manipulation is based on working memory and speed of processing and was found to improve these abilities (Horowitz-Kraus et al. 2013), it is not unexpected that the training should influence executive-function pathways in the brain. A previous study using evoked response potentials (ERP) measures with EEG found that reading improvement was associated with greater activity of the error-detection system in reading following training with the RAP in individuals with RD than TRs (in adults: Horowitz-Kraus and Breznitz 2011, in children: Horowitz-Kraus and Breznitz 2013; Horowitz-Kraus et al. 2013). The findings of the current study regarding the increased activation of the ACC in children with RD, provide complimentary spatial information to these previously electrophysiological findings. The absence of this change in TRs contradicts other studies showing a change in error detection following training with the RAP in TRs (Horowitz-Kraus and Breznitz 2011; Horowitz-Kraus and Breznitz 2013), which might suggest that the change in the error-detection activation in TRs is secondary to the change in neural circuits supporting reading (Breznitz et al. 2013; Horowitz-Kraus and Breznitz 2011; Horowitz-Kraus and Breznitz 2013). A recent study examined the differential change in ERP components in the nonlinguistic Wisconsin Card-Sorting Task and showed an improvement in attention/early perception and speed of processing abilities after the RAP in children with RD, while TRs showed only an increase in speed of processing. The authors suggested that a lower starting point of executive functions abilities in children with RD enabled them to obtain greater gains and improve a wider array of executive functions after training with the RAP (Horowitz-Kraus and Breznitz under review).

Our neuroimaging results also bear on the ongoing theoretical debate regarding the concept of fluency in the reading process. Specifically, is fluency a consequence of the ability to read single words automatically and accurately or alternatively, is it a composite of key reading-related processes (phonological, orthographical and semantic) together with more basic higher order abilities such as executive functions (attention, working memory, speed of processing) (Berninger et al. 2002; Breznitz 2006; Horowitz-Kraus et al. 2013; see also Benjamin and Gaab 2012). Here, we demonstrate that following a fluency training, we find increased activation both in phonological (STG), orthographical (IOG), semantic (IFG), and executive regions (ACC, MFG), which support the claim that fluency is a combination of both key processes related to reading and basic higher order abilities.

### Limitations of the current study

Three limitations of this pilot study should be noted. First, we used a relatively short course of the RAP training (4 weeks, 20 min per day). Previous studies have shown significant behavioral changes in reading performance after 8 weeks of one-on-one training (Denton et al. 2013). However, the current 4-week course of computer-based training is the first to report a neurobiological effect following such a short course of intervention. Presumably, a longer duration of exposure to the RAP would produce a more significant and long-lasting effect on reading scores and the correlated neural circuits. In this study, Test 2 was performed immediately after the RAP training was completed, so we were not able to assess how long the neural imprint or improvement in reading measures lasts following this short intervention course. A future study should examine the effect of a longer training period on neural circuits related to reading, as well as whether the effect of such training has a long-lasting signature on these circuits.

A second limitation of the study design is the short duration of the fMRI task for words versus pseudowords. We elected to use only 60 trials representing two conditions from the complete paradigm that consisted of four conditions (originally included also pseudohomophones and letters), to keep our focus on the contrast relevant to executive functions associated with reading. This limited the data stream in our analysis to 156 sec consisting of only 78 image volumes. Despite the limited signal-to-noise ratio in the resulting contrast maps, we found statistically significant ROI-based differences between groups and sessions that survived Bonferroni correction for multiple comparisons. Whole-brain exploratory analysis of differences in activation, either between groups or before and after training, was not possible in this case, since differences likely would not survive corrections for multiple comparisons at the voxel level. Consequently, we limited our analysis to brain regions that we a priori hypothesized would be influenced by the RAP training. These hypotheses were based on prior work with the RAP and other imaging studies in children with RD. Hypothesis-driven ROI analyses produced significant results that allowed us to explore hypotheses about neuroplastic changes in the neural circuitry of reading that correspond to normalization versus compensation strategies in the developing brain and that fit well into the context of prior literature regarding interventions for individuals with RD. Whole-brain analyses will require a future study with a larger number of subjects and longer duration neuroimaging paradigms. However, even with the short duration of training and the limited neuroimaging assessments, we have found evidence of interactions of neuroplasticity and reading improvements in children with RD in response to the RAP, providing ample evidence for further investigations into the mechanisms by which the RAP improves reading performance using DTI, fMRI, and morphometric analysis of anatomical brain images.

Third, the current study demonstrated that the effect of training with the RAP is specific to the trained group through a lack of change in reading measures in the wait-list group based on behavioral reading measures. We did note, however, that although the wait-list group did not show a significant increase in reading scores, we still did not observe a significant change between the trained or wait-list of children with RD (i.e., the absence of significant C > D contrast in Table 2). One explanation for that may be the low number of participants overall, in the two groups, and specifically in the wait-list group, resulting in larger standard deviations. A future study with a larger number of participants should clarify this point. Finally, only behavioral data were acquired from the wait-list group of children with RD. Due to the preliminary nature of the current study, we did not acquire imaging data from the wait-list group of children with RD and we did not have a wait-list group of TRs. In spite of the lack of change in reading measures in the wait-list group, this poses some limitation on the current study. A future study including behavioral and imaging data for all four conditions (TRs and children with RD in both experimental and wait-list groups), would allow the assessment of whether fMRI changes were due to training or due to repeating the fMRI task.

## Conclusions

Our study demonstrates that training with the RAP has a normalizing effect as well as a compensatory effect on neural circuits supporting reading in children with RD. The educational implications from these results are two-fold. First, they provide evidence that reading intervention programs affect neural circuits underlying reading and suggest that children may begin to use “normalized” reading pathways after as little as 4 weeks of training. This could imply that longer intervention programs may not be necessary or efficient. It is important to note that children who suffer from RD as a secondary deficit (e.g., children with ADHD) may respond differently than other children with RD and TRs to the same reading programs. Also, the greater activation of frontal regions (i.e., “compensation” pathways) in children with RD after training with the RAP supports previous findings of executive function improvement following this training (Horowitz-Kraus and Breznitz 2011; Horowitz-Kraus et al. 2013; Horowitz-Kraus and Breznitz 2013). We therefore suggest that the RAP cannot replace a one-on-one phonological processing practice, which is still needed for young children. However, the RAP could be added to establish the efficient integration of the phonological module together with the sematic and orthographic module, with the additional executive functions component. This suggests that children with RD may gain even more with a specific executive function intervention, in addition to the reading intervention. Further studies should examine these points and extend to assessment of long-term postintervention effects.
